# Microwave hyperthermia represses human papillomavirus oncoprotein activity and induces cell death due to cell stress in 3D tissue models of anogenital precancers and cancers

**DOI:** 10.1016/j.ebiom.2023.104577

**Published:** 2023-04-15

**Authors:** Michaela J. Conley, Ilaria Epifano, Anna Kirk, Andrew Stevenson, Sheila V. Graham

**Affiliations:** MRC-University of Glasgow Centre for Virus Research; School of Infection and Immunity; College of Medical, Veterinary and Life Sciences, University of Glasgow, Garscube Estate, Glasgow, G61 1QH, Scotland, UK

**Keywords:** Human papillomavirus, Cervical disease, Microwave hyperthermia, Cell stress response, HSP70

## Abstract

**Background:**

Hyperthermia is a well-accepted cancer therapy. Microwaves provide a very precise, targeted means of hyperthermia and are currently used to treat plantar warts caused by cutaneous-infective human papillomaviruses (HPVs). Other HPV genotypes infecting the anogenital mucosa cause genital warts or preneoplastic lesions or cervical cancer. Effective, non-ablative therapies for these morbid HPV-associated lesions are lacking.

**Methods:**

The molecular consequences of microwave treatment were investigated in *in vitro* cultured three-dimensional HPV-positive cervical tumour tissues, and tissues formed from HPV-infected normal immortalised keratinocytes. Microwave energy delivery to tissues was quantified. Quantitative reverse transcriptase PCR was used to quantify mRNA expression. Immunohistochemistry and fluorescence immunostaining was used to assess protein expression.

**Findings:**

Microwave energy deposition induced sustained, localised cell death at the treatment site. There was a downregulation in levels of HPV oncoproteins E6 and E7 alongside a reduction in cellular growth/proliferation and induction of apoptosis/autophagy. HSP70 expression confirmed hyperthermia, concomitant with induction of translational stress.

**Interpretation:**

The data suggest that microwave treatment inhibits tumour cell proliferation and allows the natural apoptosis of HPV-infected cells to resume. Precision microwave delivery presents a potential new treatment for treating HPV-positive anogenital precancerous lesions and cancers.

**Funding:**

Funding was through an Innovate UK Biomedical Catalyst grant (ID# 92138-556187), a 10.13039/501100000589Chief Scientist Office grant (TCS/19/11) and core support from 10.13039/501100000265Medical Research Council (MC_ UU_12014) core funding for the MRC-University of Glasgow Centre for Virus Research.

**Research in context**

**Evidence before this study**

“High risk” human papillomaviruses (HR-HPVs), cause anogenital lesions such as genital warts, anal, penile, cervical, vulval and vaginal intraepithelial neoplasia (precancers) and cancers. Incidence of most of these diseases and cancers is increasing worldwide. Laser, or electrical ablation, or “cold coagulation” (use of a hot probe to burn away lesions) are current options for treating cervical precancers and cancers. Treatment for other anogenital cancers is surgical excision. Precancerous lesions or warts may also be excised and/or treated with imiquimod (immune response modifier) or cidofovir (viral replication inhibitor). These drugs can cause inflammatory reactions, which are not well-tolerated, and lesions can recur. Women with HR-HPV-associated anal or cervical precancers are at risk of multifocal anogenital disease. This is challenging and costly to treat due to multiple lesions, significant treatment-related morbidity and sometimes the need for extensive reconstructive surgery.

Localised hyperthermia (heating to fever temperature range) has proved useful in cancer therapy to elicit anti-tumour effects, including immune activation. Microwaves can deliver mild hyperthermia in a controlled, transient, and linear fashion. Recently, microwaves were used to treat recalcitrant verrucas, caused by cutaneous-infective HPVs. Microwaving produced a 76% clearance rate, compared to, at best, 33% by cryotherapy. Data suggested that the effect was mediated through enhanced immune activation. A recent clinical trial also showed efficacy of microwaving against actinic keratoses (HPV-associated precancerous rough skin patches due to years of sun exposure). Although these dermatological diseases are caused by non-anogenital HPVs, the close genetic and biological similarity of different types of HPV suggests that heat treatment by microwaving could clear other HPV-associated diseases. Extension of therapeutic use to anogenital disease could deliver a better treatment option than current approaches. Moreover, with further development, the hyperthermia approach could be broadly applicable to other types of precancers or carcinomas *in situ*.

**Added value of this study**

This study reveals the molecular effects of microwave energy on HPV-infected epithelial tissues *in vitro*. The results show the precision of microwave delivery to tissues. Microwave energy resulted in a sustained reversal of the tumourigenic phenotype; a decrease in cellular proliferation and an increase in apoptosis/autophagy due to induction of a cellular heat shock and translational stress response. Importantly, the treatment reduced expression of the HPV oncoproteins E6 and E7, whose increased expression underlies progression of HPV-associated cancers.

**Implications of all the available evidence**

All the evidence indicates that microwave treatment using a low heat energy precision device could be a novel therapy for anogenital disease, particularly for those cases such as HPV-associated anogenital multifocal disease that normally prove difficult to treat effectively. Current treatment for anogenital disease can cause sustained bleeding or inflammation and diseased tissue can be missed. Heat treatment through microwaving could provide better tissue coverage and be more acceptable and better tolerated than existing strategies. The mild nature of the procedure would mean that sequential treatments could be spaced more closely and could save clinic time and resources.

## Introduction

Around 40 human papillomaviruses (HPVs) infect the anogenital region.^1^ Some cause genital warts but others (“high-risk” (HR-HPV)) cause >99% of cervical cancers and between 40 and 90% of other anogenital cancers in men (anal, perineum, penile) and women (anal, vulvar, vaginal, perineum).^2^^,^^3^ Incidence of these cancers is increasing.^3^ Treatment of cervical precancers, or cervical intraepithelial neoplasia (CIN), is “loop” excision (LLETZ) of the cervix^4^ or “cold coagulation”, cauterisation with a heated probe.^5^^,^^6^ These treatments are effective but invasive and painful, and for LLETZ, often with subsequent bleeding and a two to three-fold increase in preterm birth.^7^ Treatment of other anogenital precancers also involves excision.^4^ Those with HR-HPV-associated anal or cervical precancers are at risk of multifocal anogenital disease,^8^ which is challenging and costly to treat with significant morbidity due to treatment and the need for a multidisciplinary clinical team for disease management.^4^ In all cases, diseased tissue can be missed. A new, less invasive and painful method for treating HPV-associated anogenital disease and cancers could prove more acceptable and better tolerated by patients than current procedures and would save time and resources for clinicians and health care systems.

Hyperthermia, especially in combination with radio- or chemotherapies, is a well-known treatment for cancers such as cervical, melanoma and breast cancer.^9^, ^10^, ^11^ Sustained temperatures above 40 °C result in membrane disruption, DNA and protein damage and cell cycle arrest followed by necrosis or apoptosis.^12^ Heating causes cytotoxicity through impaired functions of proteins involved in key cellular processes such as DNA replication and can induce anti-tumour immune responses.^10^

Microwaves efficiently deliver mild heat energy (<50 °C) in a highly controlled, transient localised, linear manner.^13^ They produce non-ionising radiation, so do not induce DNA damage. The delivery of microwaves to tissues reaches a depth of up to 6 mm depending on the generator frequency and energy level used and there is little lateral spread. Microwaves generate heat by a process known as dielectric hysteresis. Water molecules in tissues try to align with an electromagnetic field of rapidly alternating polarity generated by microwaves. This results in rapid spinning of water molecules in the tissue and this energy is converted to rapid, highly localised heat generation. Thus, heat generated by microwaves is quite different from heat generated by other means particularly since it involves heating from the inside to the outer surface.^14^ This method of heating has been used previously to treat cancers such as liver cancer.^15^

A CE-marked portable medical device which delivers microwaves through a 6.7 mm contact site is currently approved for use in in the fields of podiatry and dermatology in the treatment of plantar warts (verrucas).^16^ Plantar warts are caused by infection with cutaneous-infective human papillomaviruses (HPVs), e.g. HPV genotype 1.^17^ Microwave treatment of plantar warts resulted in the shrinkage and clearance of lesions without significant inflammation, visible tissue damage or scarring and an antiviral immune response was generated.^18^ There was a final resolution rate of 75.9% compared to 23–33% for standard cryotherapy treatments. Low pain scores were reported which decreased as treatment plans proceeded.^16^

A recent UK clinical trial (NCT03483935) showed microwave therapy to be a safe and effective therapy for actinic keratoses,^19^ which may have an underlying beta-HPV aetiology.^20^ Although these types of cutaneous lesions are caused by non-anogenital-infective HPVs, the close genetic and biological similarity of different types of HPV suggests that heat treatment by microwave energy could clear other HPV-associated diseases.

In this study we tested if localised microwave hyperthermia could have a therapeutic effect against HPV-associated anogenital precancerous and cancerous tissues. For the cancer model we used SiHa cells, which are HPV16-positive cervical epithelial cells derived from a grade II squamous cell carcinoma.^21^ The precancer model was normal immortalised foreskin keratinocytes expressing the HPV16 genome (NIKS16) or the HPV18 genome (NIKS18).^22^^,^^23^ To mimic *in vivo* tissue, cells were grown in three dimensional organotypic raft cultures.^24^ We determined the energy settings required to elevate tissue temperature to 45–48 °C. Application of microwaves caused local tissue heating and there was reduced cell proliferation at adjacent sites concomitant with expression of apoptosis markers. HSP70 was induced indicating successful temperature elevation. Microwave-treated cells displayed a translation stress response. Importantly, in SiHa cancer cells HPV oncoprotein expression was reduced. Correlating with this, levels of the apoptosis regulator p53, normally repressed by HPV E6^25^ and levels of Rb, normally a degradation target of HPV E7^26^ were significantly increased in the tissues upon microwave treatment. Taken together the data suggest that microwave treatment of HPV-positive tumour tissue reversed the tumour phenotype and induced cell stress leading to inhibition of tissue growth and induction of apoptosis.

## Methods

### Cell lines

J2 3T3 fibroblasts were grown in Dulbecco's modified eagle medium (DMEM; Life Technologies) supplemented with 10% donor calf serum, 2 mM l-Glutamine (Life Technologies), 100 units/ml penicillin and 100 μg/ml streptomycin (PenStrep, Life Technologies).^24^ NIKS16 (RRID:CVCL_B0UM) and NIKS18 (RRID:CVCL_B0UP) cells were grown on 3T3 fibroblast feed layers in E-medium as previously described.^27^ SiHa cells (IZSLER Cat# BS TCL 112, RRID:CVCL_0032)^21^ were grown in DMEM supplemented with 10% foetal bovine serum (FBS), 100 units/ml penicillin and 100 μg/ml streptomycin (PenStrep, Life Technologies). All cells were cultured at 37 °C in 5% carbon dioxide (CO_2_).

### Cell line validation

All cell lines were routinely tested for mycoplasma. SiHa, NIKS16 and NIKS18 cell lines were validated by routinely comparing morphology from batch-to-batch growth in 2D and 3D tissue cultures and by quantifying HPV DNA genome numbers, and E6E7 mRNA expression levels by qRT-PCR. Expression of viral life cycle markers E4 and L1 proteins was also verified in each batch of NIKS16 or NIKS18 cells to ensure they supported HPV replication.^28^ NIKS16 and NIKS 18 cell lines were used at passage <12 to avoid HPV genome integration. Tissue distribution of proliferation and differentiation markers was validated as unchanged in every experiment by examining in-tissue expression of MCM2, Ki67, Involucrin and Keratin 10 in untreated tissues by immunofluorescence and western blotting.

### Antibodies

Immunohistochemistry (IHC) antibodies were: active caspase 3 (R and D Systems Cat# AF835, RRID:AB_2243952, 1:1000), mini chromosome maintenance protein 2 (Abcam Cat# ab31159, RRID:AB_881276, 1:100), Ki67 (Agilent Cat# M7240, RRID:AB_2142367, 1:200), p53 (Abcam Cat# ab1101, RRID:AB_297667, 1:3000), Rb (Cell Signaling Technology Cat# 9309, RRID:AB_823629, 1:1600) and heat shock protein 70 (HSP70) (Sigma–Aldrich Cat# H5147, RRID:AB_477057, 1:3200). Immunofluorescence antibodies were used at the following dilutions: HPV E6 (Euromedex, #6F4, 1:200), HPV E7 (Thermo Fisher Scientific Cat# 28-0006, RRID:AB_2533057), 1:50), cleaved caspase 3 (R and D Systems Cat# AF835, RRID:AB_2243952, 0.3 μg/ml), Ki67 (Abcam Cat# ab206633, RRID:AB_2861195, 1:1000) GTPase-activating protein SH3 domain-binding protein (G3BP) ((Abcam Cat# ab56574, RRID:AB_941699, 1:250) and poly (A) binding protein C1 (PABPC1) (Abcam Cat# ab21060, RRID:AB_777008, 1:1000), Keratin 10 ((Abcam Cat# ab9026, RRID:AB_306950, 1:200), MCM2 (Abcam Cat# ab31159, RRID:AB_881276; 1:100), involucrin (Sigma–Aldrich Cat# I9018, RRID:AB_477129, 1:200), LC3B (Novus Cat# NB100-2220, RRID:AB_10003146, 1:200), p62 (MBL International Cat# PM045B, RRID:AB_1953130: 1:500), and HSP70 (Sigma–Aldrich Cat# H5147, RRID:AB_477057), 1:200).

### Antibody validation

Antibody validation was carried out by examining protein detection at a range of antibody concentrations and quantifying reproducibility between experiments. Specificity was examined by carrying out western blotting to ensure that a single band of the correct molecular mass was obtained with each antibody. Secondary antibody only controls were included in every experiment. E6 antibody is not suitable for western blotting and so was validated by comparing reactivity in immunofluorescence in HPV-negative C33a cells and C33a cells stably expressing E6.^29^ Other antibodies were verified by comparing protein depletion following siRNA knock down by western blotting. The same batch of each antibody was used throughout.

### 3D organotypic raft tissue cultures

3D cultures were performed exactly as described,^24^ grown on metal grids and incubated at 37 °C with 5% CO_2_ at the air liquid interface with E-medium for 14 days to allow tissue growth. E-medium was composed of a three to one ratio of DMEM: F12 medium (Life Technologies) supplemented with 10% FBS, 100 units/ml penicillin and 100 μg/ml streptomycin (PenStrep, Life Technologies) 2 mM l-glutamine, 180 μM adenine, 5 μg/ml transferrin, 5 μg/ml insulin, 0.4 mg/ml hydrocortisone, 0.1 nM cholera toxin and 0.2 ng/ml epidermal growth factor (EGF).^24^

### Microwave treatment of 3D tissue cultures

3D *in vitro* grown tissues were lifted from the grids and placed cell side down onto the lid of a 10 cm dish that had been pre-incubated at 37 °C (Supplementary Figure S1). The dish was closed, and the microwave probe was then positioned underneath the plastic lid, facing the cell layers of the tissue and set to deliver 10 W of power for 10 s (unless otherwise specified). Tissues were either fixed immediately in formalin overnight or returned to the air-liquid interface and incubated for up to 144 h at 37 °C in 5% CO_2_ prior to fixation. “Mock treated” tissues underwent the same procedure but were not subject to microwave energy.

### Temperature measurements of organotypic raft cultures

A temperature probe (NOMAD-Touch, by Neoptix, Canada) was used to measure the temperatures of the rafts by placement on the surface of the tissues during the microwave treatment (Supplementary Figure S1).

### Immunohistochemistry

Formalin fixed tissue cultures were paraffin-embedded and sectioned. Sections (2.5 μm) were stained with haematoxylin and eosin (H&E) or for antibody staining were subject to heat induced epitope retrieval (HIER). HIER methods were sodium citrate buffer pH 6 for all antibodies except p53 where treatment with EDTA buffer was carried out at pH 9. Sections were subsequently stained with the appropriate antibodies at the Veterinary Pathology Laboratory, University of Glasgow. Sections were imaged on an Olympus Bx57 microscope using either ×4, ×10 or ×20 lenses.

### Immunofluorescence microscopy

3D tissues were fixed, embedded, sectioned and subjected to heat antigen retrieval prior to blocking in 10% donkey serum for 1 h at room temperature (RT). Slides were washed in phosphate buffered saline (PBS) and incubated in 5% donkey serum containing the primary antibody at 4 °C for 2 h or overnight. Slides were subsequently washed in PBS and incubated in 5% donkey serum containing the appropriate fluorescently labelled (AlexaFluor 488 or 555) secondary antibody (ThermoFisher Scientific) at RT for 1 h. Slides were washed in PBS and deionised water and coverslips mounted using ProLong Gold Antifade reagent with DAPI (ThermoFisher Scientific). Slides were visualised on a Zeiss LSM880 laser scanning microscope or on an LSM710 using ZEN 3.2 Blue software and fluorescence was quantified using ImageJ.

### RNA extraction and cDNA synthesis

RNA was prepared from 3D tissues using QIAzol Lysis reagent and the RNeasy kit (Qiagen) according to the manufacturer's instructions. RNA concentration was determined using a Nanodrop One/One Microvolume UV–Vis Spectrophotometer (Thermofisher). cDNA was synthesised from total RNA (500 ng) using the Maxima First Strand cDNA Synthesis Kit for RT-qPCR with DNase, according to the manufacturer's instructions.

### Quantitative reverse transcriptase PCR (qRT-PCR)

Primers for the HPV16 bicistronic E6E7 transcript and the housekeeping gene β-actin, which was used as a reference, were designed using PrimerQuest (Integrated DNA Technologies). E6E7 Forward primer: 5′-CAATGTTTCAGGACCCACAG-3′, E6E7 reverse primer: 5′-CTGTTGCTTGCAGTACACACATTC-3′, E6E7 probe: 5′-CCACAGTTATGCACAGAGCTGC-3’. Beta-actin forward primer: 5′-AGCGCGGCTACAGCTTCA-3′, beta-actin reverse primer: 5′-CGTAGCACAGCTTCTCCTTAATGTC-3′, beta-actin probe: 5′-ATTTCCCGCTCGGCCGTGGT-3’. Quantitative Real-Time PCR was performed using a 7500 Real Time PCR System (Thermofisher). Each qRT-PCR reaction (total volume of 20 μl) included 10 μl of Takyon ROX Probe 2× MasterMix dTTP blue (Eurogentec), 4 μl of primer/probe mix (final concentrations of 900 nM and 100 nM for primers and probes respectively). 5 μl of cDNA was added. Reaction conditions were one cycle at 50 °C for 2 min, one cycle of 95 °C for 3 min followed by 40 cycles of 95 °C for 10 s followed by 60 °C for 1 min. Each sample was assayed in triplicate. Data produced in each qPCR reaction was analysed on the 7500 Real-Time SDS Software (Thermofisher). The threshold line for C_T_ determination was assigned automatically and was always within the exponential phase. Relative quantification of viral mRNA was done using the Livak method (2^−ΔΔCT^).

### Statistical analysis

All experimental data shown are representative of at least three individual experiments. Statistical analysis was performed with GraphPad Prism 7 using a student's T-test or for comparison between groups, an ANOVA Kruskal–Wallis test.

### Role of the funding source

This work was funded by an Innovate UK Biomedical Catalyst grant (ID# 92138-556187), and by a Chief Scientist Office grant (TCS/19/11). We acknowledge support from the Medical Research Council (MC_ UU_12014) as core funding for the MRC University of Glasgow Centre for Virus Research. None of the funding sources had any input into study design, the collection, analysis, and interpretation of data, the writing of the report or the decision to submit the paper for publication. All authors are employed by the University of Glasgow apart from A. Kirk who is a PhD student on an MRC-funded PhD programme at the University of Glasgow. Emblation Ltd provided the medical device but had no input into the study design, the collection, analysis, and interpretation of data, the writing of the report or the decision to submit the paper for publication.

## Results

**Fig. 1:**
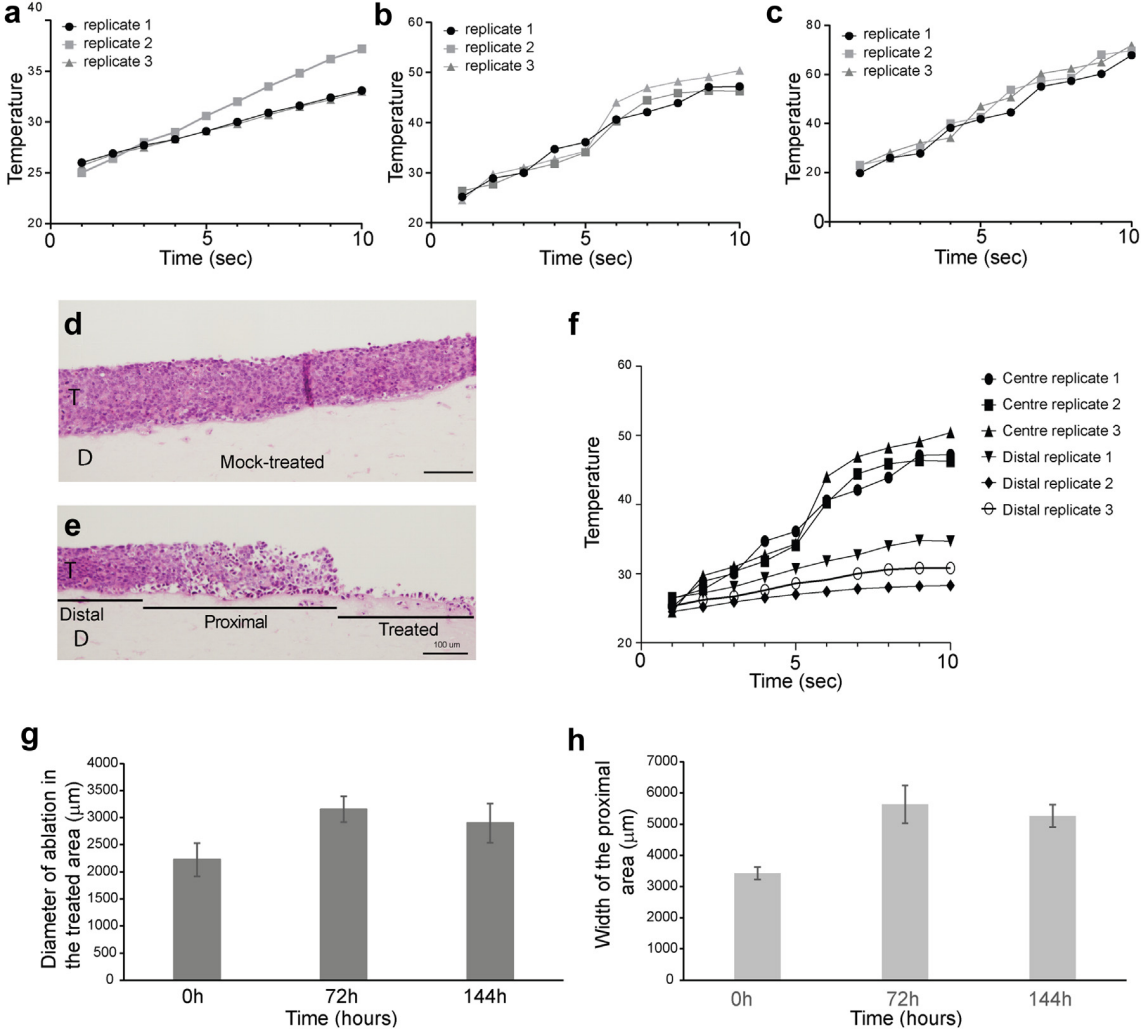
**The effect of microwave treatment on three dimensional SiHa tissues.** Organotypic raft cultures of SiHa cells were grown at the air-liquid interface for 14 days then treated with microwaves at (a). 5 W, (b). 10 W, or (c). 15 W for up to 10 s. The graphs show the measurements of temperature increase every second over the 10 s time period in three independent tissues (replicates 1, 2 and 3) (d). H&E-stained section of a mock-treated formalin-fixed and paraffin-embedded (FFPE) SiHa tissue. (e). H&E stained FFPE section of a SiHa tissue treated with microwave energy at 10 W for 10 s. Both tissues were harvested immediately after treatment. The areas of the treated tissue we have designated as “treated” (cell disruption), “proximal” (loss of cell–cell contact) and “distal” (no significant tissue disruption) are indicated. T = SiHa tissue. D = collagen/fibroblast dermal equivalent. Scale bars in (d) and (e) =100 μM. (f). Graph of measurement of temperature increase every second over a 10 s period comparing temperature at the centre of the tissue (taken from Fig. 1(b)) with temperature at a distal portion of the same tissue in three replicate tissues (distal replicates 1, 2 and 3). A diagram of the experimental set-up is shown in Supplementary Figure S1(d). (g). Graph of the average diameter of the treated area of SiHa tissues over a 6-day period of re-incubation at 37 °C after microwave treatment. (e). Graph of the average diameter of the proximal area of SiHa tissues over a 6-day period of re-incubation at 37 °C after microwave treatment. Data in (g) and (h) show the average and standard error of the mean from five separate experiments.

We adopted a three-dimensional (3D) epithelial raft culture system^24^ to study the molecular impact of microwave treatment on cervical tumours *in vitro*. SiHa cells are derived from a grade II cervical squamous cell carcinoma.^21^ They contain two integrated copies of the HPV16 genome per cell^30^ and express HPV16 E6 and E7 oncoproteins.^31^ Unlike other cervical cancer cell lines,^32^ SiHa cells form undisrupted, non-invasive multi-layered undifferentiated tissues in 3D cultures when grown at the air-liquid interface,^32^ making them a useful model for this study (Fig. 1d).

### Optimisation of microwave treatment

A range of treatment conditions were tested for temperature elevation in the SiHa 3D tissues due to microwave treatment: 5 W, 10 W or 15 W for time periods up to 40 s. Microwaves were applied to the SiHa tissues by inverting them onto the lid of a preheated plastic cell culture dish with the microwave device located on top of the tissue (Supplementary Figure S1). Triplicate temperature increase curves over a 10 s period are shown for 5 W (Fig. 1a) 10 W (Fig. 1b) or 15 W (Fig. 1c) treatments. Treatment with 10 W for 10 s resulted in the target temperature increase of between 45 and 48 °C (Fig. 1b). This thermal envelope and transient time is consistent with that of the tissues in plantar warts under an optimised protocol in use clinically, verified by in silico modelling using Multiphysics finite element analysis software (Comsol, Sweden) by the microwave device manufacturer. Compared to mock-treated SiHa tissue (Fig. 1d), treatment at 10 W for 10 s resulted in an immediate and discrete damage of the tissue at the treatment site (Fig. 1e). In the tissue areas proximal to the treatment site tissue integrity was compromised with gaps visible between cells, but away from the treated area the tissue appeared undisrupted. There was a clear demarcation between the treatment site and the untreated area, as observed *in vivo* in the plantar wart study.^18^ We designated three tissues areas: “treated”–the area under the microwave probe, “proximal”–the adjacent area of disrupted tissue integrity and “distal”–undisrupted tissue (Fig. 1e). For a quantitative portioning of the treated tissue, we measured temperature change over 10 s at the centre (“treated” area) and distal areas of the tissues using two separate temperature probes (Supplementary Figure S1). A temperature increase of between 45 and 50 °C within 10 s was detected in the central portion of the tissue. In the distal portion, temperature only reached 32 °C after 10 s (Fig. 1f) demonstrating spatial precision of microwave energy treatment. The entire 3D tissue has a diameter of 14 mm. The 6.7 mm applicator head of the microwave device gave an average treatment area of 2.2 mm (±0.3 mm) diameter at the “treatment site”, which was increased over time to around 3 mm diameter due to cell death (Fig. 1g). The disrupted “proximal” region measured an average width of 3.4 mm (±0.3 mm), which also increased over time to a maximum of 5.5 mm (Fig. 1h).

**Fig. 2:**
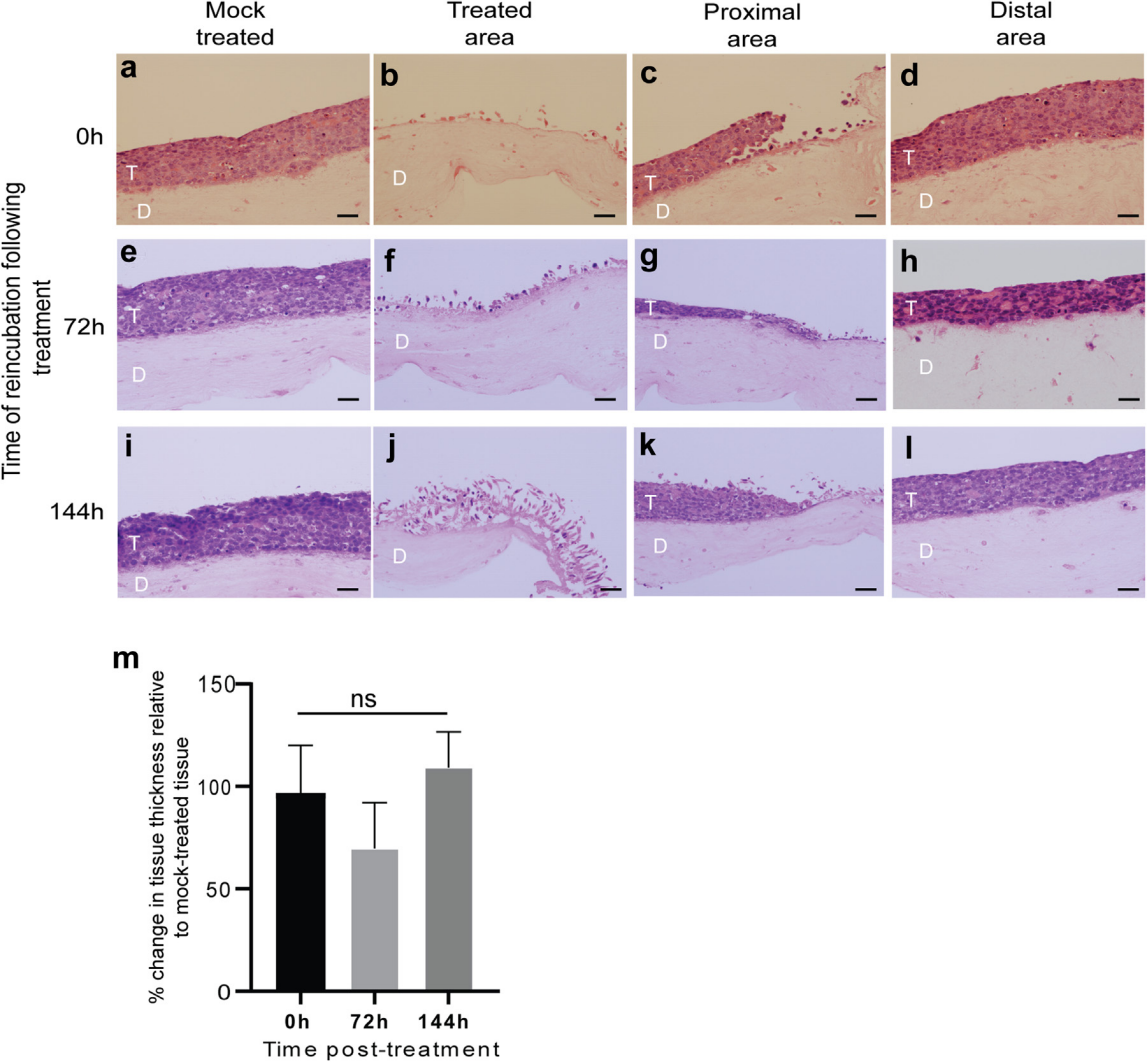
**Changes in growth of SiHa tissues due to microwave treatment over a 6-day period.** (a), (e), (i), mock-treated tissues re-incubated at 37 °C for 0, 72 or 144 h respectively following mock treatment. (b), (f), (j), images of the treated area of microwave-treated tissues re-incubated at 37 °C for 0, 72 or 144 h respectively following treatment. (c), (g), (k), images of the area proximal to the treated area of microwave-treated tissues re-incubated at 37 °C for 0, 72 or 144 h respectively following treatment. (d), (h), (l), images of the area distal to the proximal area of microwave-treated tissues re-incubated at 37 °C for 0, 72 or 144 h respectively following treatment. All images show H&E-stained 2.5 μm sections of FFPE *in vitro*-grown SiHa tissues. T = SiHa tissue. D = collagen/fibroblast dermal equivalent. Scale bars = 50 μM. The images are representative of five separate experiments. (m). Graph of changes in 3D tissue thickness at areas distal to the treated area at 0, 72 and 144 h post-microwave treatment. The data show the average and standard deviation from the mean from three separate experiments. ns = not significant p > 0.05 (student's t-test).

Fig. 2 shows haematoxylin and eosin (H&E)-stained images of treated, proximal and distal areas of tissues that were microwave-treated then re-incubated at 37 °C for up to 6 days to examine any tissue regrowth. This is the longest time period (20 days in total) tissue growth can be supported in 3D culture. Treated areas of the tissues did not regrow over this time (Fig. 2 compare image b and j). In the tissue areas proximal to treatment, growth was reduced at 72 h (Fig. 2g), but cells regrew after 144 h (Fig. 2k). In the distal areas, away from the treatment site (Fig. 2d, h, l) there was a reduction in tissue thickness at 72 h suggesting growth inhibition or ongoing cell death due to microwave treatment (Fig. 2m). However, tissue thickness recovered to a level very similar to 0 h treated tissue indicating that tissue growth was not permanently inhibited (Fig. 2m).

### Microwave treatment reduces HPV-16 oncoprotein levels and re-introduces p53 and Rb expression

**Fig. 3:**
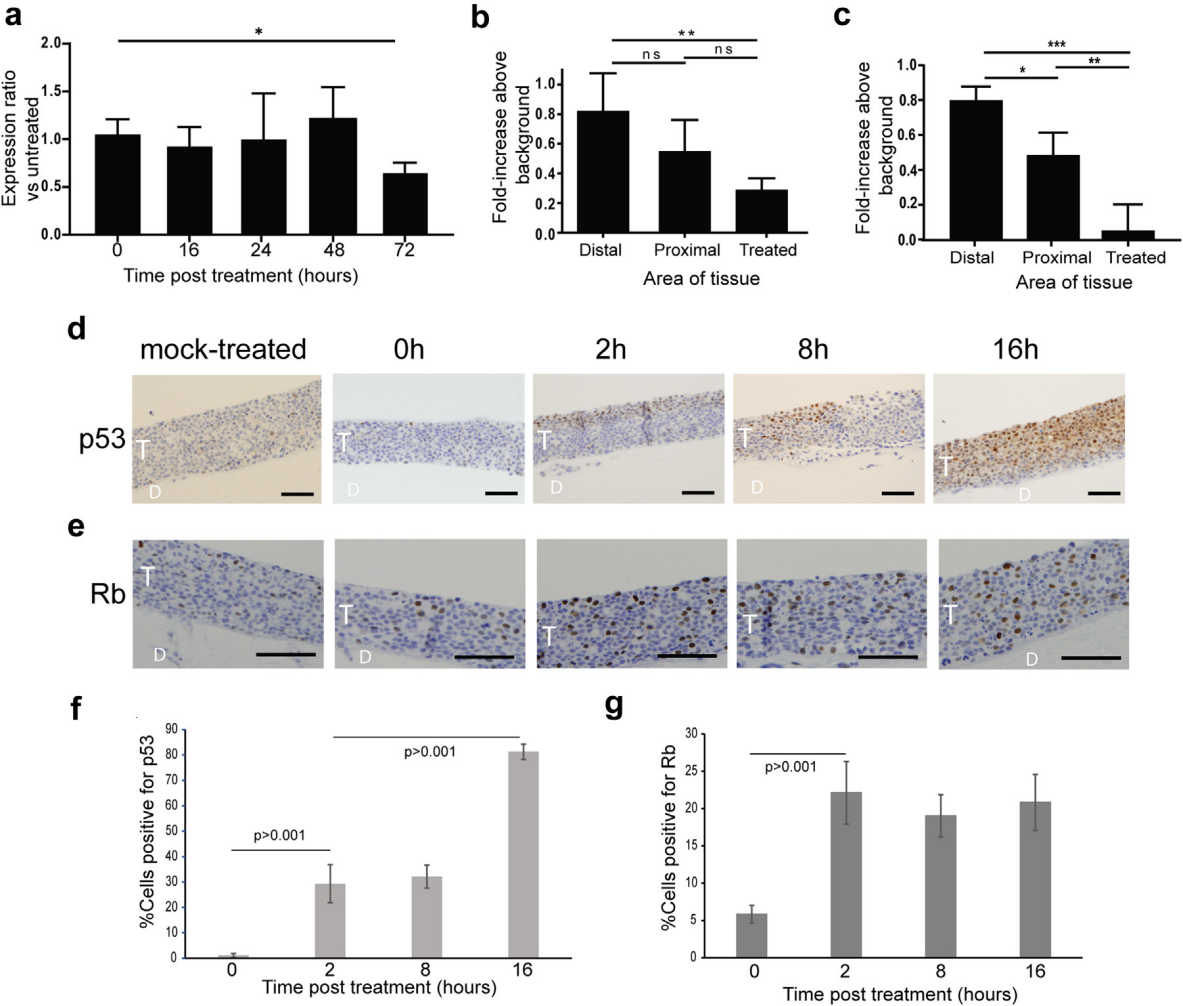
**Microwave treatment reduces HPV oncoprotein levels and re-introduces p53 and Rb expression.** (a). “Mini” 3D SiHa tissues of 7 mm diameter were microwave-treated with 10 W for 10 s and either fixed immediately (0hr) or re-incubated at the air-liquid interface at 37 °C for 16, 24, 48 or 72 h prior to harvesting for RNA extraction. Following cDNA synthesis, levels of E6/E7 bicistronic mRNA were quantified by RT-qPCR. Data are expressed as changes in E6/E7 mRNA levels relative to changes in beta-actin mRNA levels (ΔΔCt) and relative to levels in mock-treated tissues. The data shown are the mean and standard deviation from the mean of three separate experiments using three independent tissues. ∗p < 0.05 (student's t-test). Sections of microwave-treated tissues were immunofluorescence stained with an antibody against (b) HPV16 E6, or an antibody against (c) HPV16 E7. Immunofluorescence intensity was quantified using ImageJ in 50 individual cells in the treated, proximal and distal areas of three separate 3D SiHa tissues. (b). Graph of E6 protein levels in the distal, proximal and treated areas of SiHa tissues that were microwave-treated then reincubated for 16 h. (c). Graph of E7 protein levels in the distal, proximal and treated areas of SiHa tissues that were microwave-treated then reincubated for 16 h. The graphs show the mean and standard deviation from the mean using data from three separate experiments. ∗p < 0.05, ∗∗p < 0.01, ∗∗∗p < 0.001 (student's t-test). (d). Immunohistochemistry staining with an antibody against p53 of SiHa tissues mock-treated or microwave-treated and reincubated for 0, 2, 8 or 16 h. (e). Immunohistochemistry staining with an antibody against Rb of SiHa tissues mock-treated or microwave-treated and reincubated for 0, 2, 8 or 16 h. Cell nuclei are counterstained with haematoxylin (blue stain). T = SiHa tissue. D = collagen/fibroblast dermal equivalent. Data shown are representative of three individual experiments. Scale bars = 50 μm. (f). Graph showing quantification of percentage cells positive for p53 protein in tissues at 0, 2, 8, and 16 h following microwave treatment. Percentage positively stained cells in three replicate tissues were quantified by ImageJ. At least 150 cells in each of five replicate tissues were counted for each time group. p-values were determined by student's t-tests. (g). Graph showing quantification of percentage cells positive for Rb protein in tissues at 0, 2, 8, and 16 h following microwave treatment. Percentage positively stained cells in three replicate tissues were quantified by ImageJ. At least 150 cells in each of 6 replicate tissues were counted for each time group. Graphs show the average and standard deviation from the mean. p-values were determined by student's t-tests.

Cervical tumour progression is caused by increased expression of the HPV oncoproteins E6 and E7 and their repressive effects on p53 and Rb respectively. Therefore, next we measured levels of E6 and E7-encoding mRNAs. For this experiment, because we harvested RNA from the entire 3D tissue, we grew “mini” 3D tissues of 7 mm diameter to ensure that most cells in the tissue were exposed to microwave energy. Quantitative RT-PCR was carried out on cDNA synthesised from replicate RNA preparations from replicate tissues to detect E6/E7 bicistronic mRNA. No change was detected in E6/E7 mRNA expression in response to microwave treatment for the first 48 h following treatment (Fig. 3a). However, at 72 h there was a statistically significant decrease (p < 0.05) in E6/E7 mRNA expression. Next, we measured changes in E6 and E7 protein expression due to microwave energy. Following microwave treatment, 3D tissues were re-incubated at the air-liquid interface at 37 °C for 16 h prior to immunofluorescence staining with antibodies directed against E6 or E7. Fluorescence levels in 50 individual cells from treated, proximal and distal areas of three separate treated rafts were quantified (Fig. 3b and c). For both viral oncoproteins, a statistically significant reduction in fluorescence was detected in cells remaining in the treated area when compared to distal areas of the tissues. Although less fluorescence was also detected in the area proximal to treatment, this was found to be statistically significant only for E7 protein.

Next, microwave-treated tissue sections were stained for the presence of the tumour suppressor and apoptosis activator, p53. E6 forms a complex with E6 associated protein (E6AP) and p53 to target p53 for proteasomal degradation.^33^ Fig. 3d shows images of microwave-treated tissues (proximal area) which have been re-incubated at 37 °C for various times following treatment. A low level of p53 expression was detected at 0 h post treatment. At 2 h post-treatment increased p53 levels (26.7-fold increase) were apparent in the upper layers of the tissue and p53 levels increased (74.0-fold increase) up to 16 hrs post treatment. At this time, almost all the cells appeared positive for p53. High risk HPV E7 protein degrades the cell cycle check point inhibitor Rb.^26^ Due to reduced E7 levels, increased Rb levels (3.8-fold increase) were detected within 2 h post-microwave treatment and this was sustained over a 16 h period (Fig. 3e). Quantification showed that the observed increases in p53 and Rb levels were statistically significant (Fig. 3f and g). Taken together, these findings suggest that microwave treatment reduces the expression of HPV E6 and E7 oncoproteins, concomitant with an increase in expression of p53 and Rb.

### Microwave treatment reduces cellular proliferation and induces apoptosis

The observed increase in p53 and Rb levels would allow for apoptosis to resume while inhibiting cell proliferation. Next, we used immunohistochemistry staining to test this directly.

**Fig. 4:**
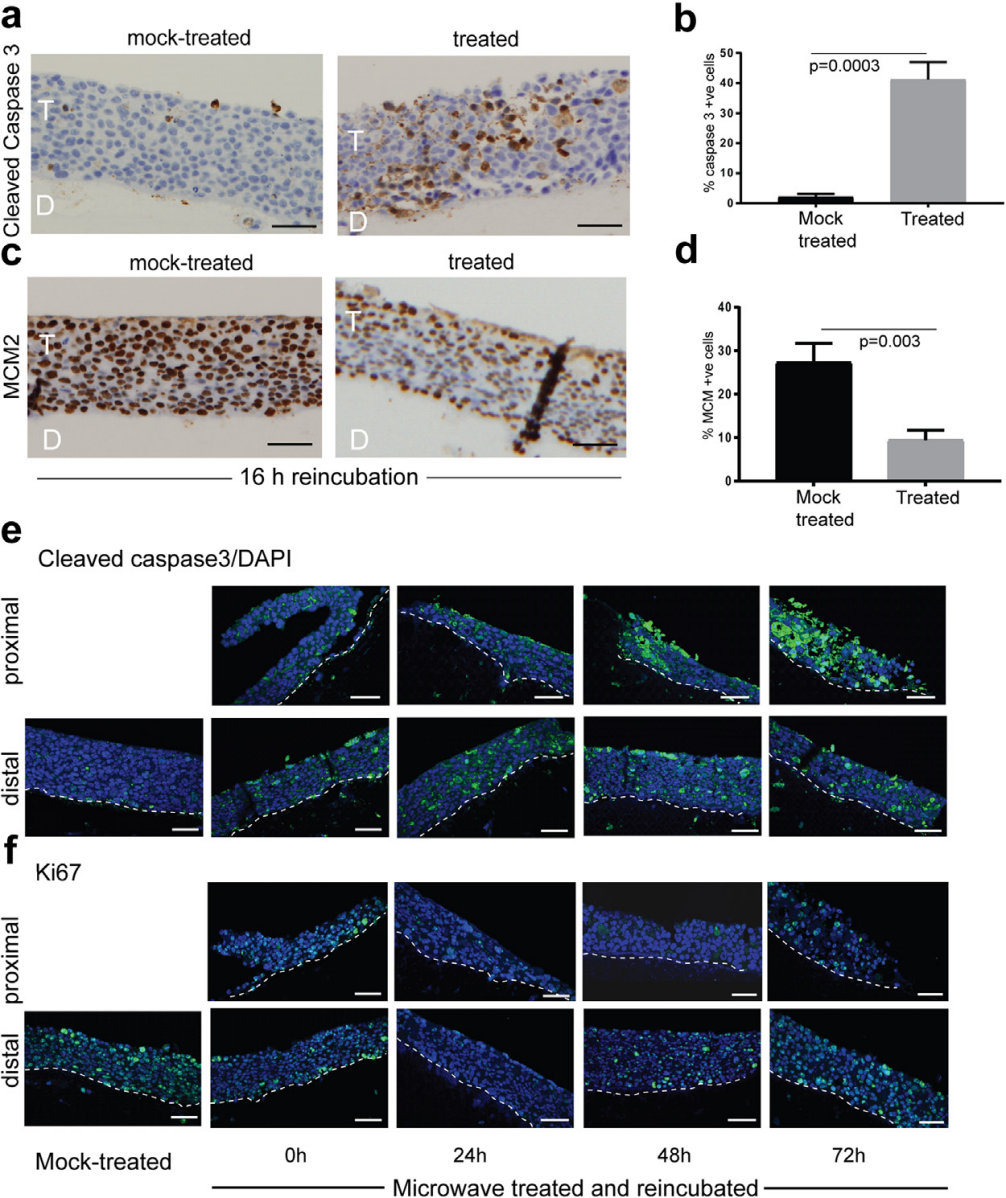
**Microwave treatment induces apoptosis and reduces cellular proliferation.** SiHa tissues were mock-treated or microwave-treated with 10 W for 10 s then re-incubated at 37 °C for 16 h. (a) 2.5 μm sections of FPPE tissues were immunohistochemistry stained with an antibody against cleaved caspase 3 (apoptosis marker). Brown staining indicates the presence of the protein of interest. Nuclei are stained blue by haematoxylin. T = SiHa tissue. D = collagen/fibroblast dermal equivalent. Scale bars = 50 μm. (b). Graph of gain of cleaved caspase 3 staining in microwave-treated tissues compared to mock-treated tissues. Percentage positively stained cells in three replicate tissues was quantified by counting stained nuclei in at least 150 cells in three replicate tissues. The graph shows the average and standard deviation from the mean. p-value was determined by student's t-test. (c) 2.5 μm sections of FPPE tissues were immunohistochemistry stained with an antibody against MCM2 (cellular proliferation marker). Brown staining indicates the presence of the protein of interest. Nuclei are stained blue by haematoxylin. The thick black lines in the treated tissue are caused by tissue fold-over at these points. T = SiHa tissue. D = collagen/fibroblast dermal equivalent. Scale bars = 50 μm. (d). Graph of loss of MCM2 staining in microwave treated tissues compared to mock-treated tissues. Percentage positively stained cells in three replicate tissues were quantified by counting stained nuclei in at least 150 cells in three replicate tissues. The graph shows the average and standard deviation from the mean. p-value was determined by student's t-test. (e). Cleaved caspase 3 immunofluorescence staining (green staining) of mock-treated or microwave-treated tissues re-incubated at 37 °C for the indicated times following treatment. (f). Ki67 (cellular proliferation marker) immunofluorescence staining (green staining) of mock-treated or microwave-treated tissues re-incubated at 37 °C for the indicated times following treatment. Nuclei are counter stained with DAPI (blue). White dotted lines show the basal layer of the tissues and junction with the dermal equivalent. Scale bars = 50 μm. Data shown are representative images from three separate experiments.

Mock microwave treatment was compared to treatment at 10 W for 10 s, followed by re-incubation at 37 °C for 16 h. Cleaved caspase 3 staining of treated tissue sections revealed induction of apoptosis only in microwave-treated tissues (Fig. 4a). Quantification of caspase 3 levels showed significantly increased expression in treated tissues compared to mock-treated tissues (Fig. 4b). The opposite effect was seen in tissues stained for a marker of cellular proliferation, MCM2 (mini chromosome maintenance protein 2) (Fig. 4c), with significantly fewer positive cells being detected in the microwave-treated tissue (Fig. 4d). Increased cleaved caspase 3 expression in microwave-treated tissues was sustained over 72 h, especially in the proximal tissue regions (Fig. 3e). Ki67, a second marker of cell proliferation, was repressed up to 24 h post-treatment in both proximal and distal areas of treated tissues (Fig. 3f). Some increase in cell proliferation was observed in the distal areas between 48- and 72-h following treatment compared to time periods up to 24-h post-treatment. Our results show that treatment with microwave energy resulted in induction of apoptosis as well as an inhibition of cellular proliferation i.e., a reversal of the tumour phenotype.

### Microwave treatment induces a heat shock response

**Fig. 5:**
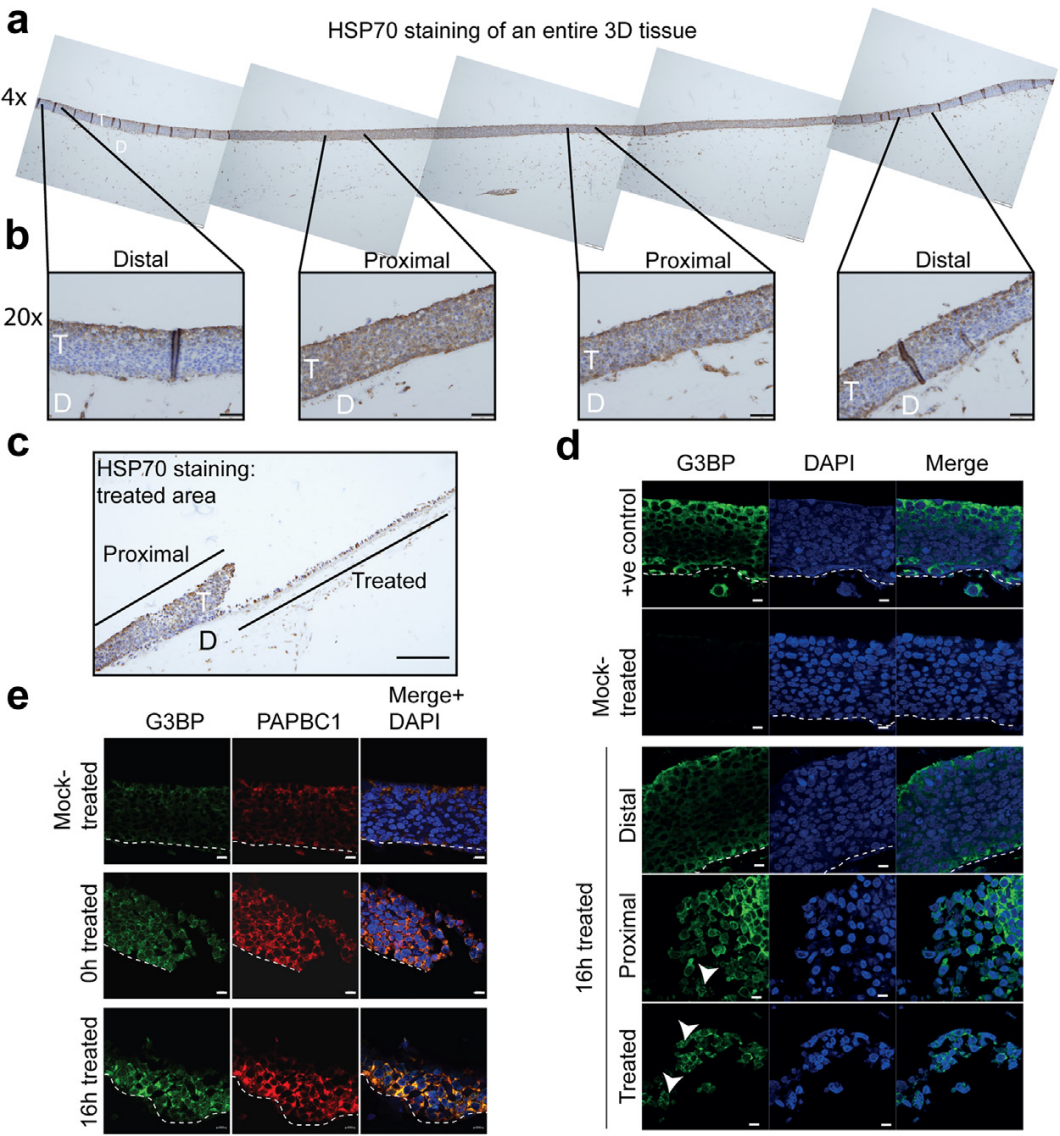
**Microwave treatment induces a cellular stress response.** (a). 4× magnified image of an entire 3D SiHa tissue microwave-treated and re-incubated at 37 °C for 16 h. Immunohistochemistry staining for HSP70 expression is shown. (b). 20× magnified panels of two distal and two proximal regions from the tissue in (a). T = SiHa tissue. D = collagen/fibroblast dermal equivalent. No “treated” region is visible because the 3D tissue has been sectioned behind the zone of tissue disruption. Scale bars = 50 μM. (c). Immunohistochemistry staining for HSP70 of a tissue microwave-treated and re-incubated at 37 °C for 16 h showing the treated and the proximal areas. T = SiHa tissue. D = collagen/fibroblast dermal equivalent. Scale bar = 50 μM. (d) Immunofluorescence staining for G3BP (green staining) was performed on heat-shocked (+ve control: incubated at 40 °C in culture for 6 h), mock-treated, and microwave-treated SiHa tissues which had been re-incubated at 37 °C for 16 h. Staining in distal, proximal and treated areas of a treated tissue is shown. Arrowheads indicate G3BP-positive cytoplasmic granules. (e). Immunofluorescence co-staining with antibodies against G3BP (green staining) and PABPC1 (red staining) of mock-treated or microwave-treated SiHa tissues fixed immediately following treatment (0 h) or re-incubated at 37 °C for 16 h. Blue staining = DAPI. White dotted lines show the basal layer of the tissues and junction with the dermal equivalent. Scale bars = 10 μm. Data shown are representative images from three separate experiments.

Heating to fever temperature (>38 °C) and above induces thermal stress, which can be reversible or can result in cell death. Microwave therapy has previously been shown to induce heat shock protein expression in skeletal muscle.^34^ To determine if a heat shock response was induced in the 3D tissues following microwave treatment, tissues (treated and re-incubated for 16 h) were immunohistochemically stained for the molecular chaperone HSP70, whose expression is known to create a thermotolerant cellular environment.^35^ An entire HSP70-stained tissue section is shown in Fig. 5a. Due to the cutting of the section away from the site of direct microwave treatment site, no area devoid of cells is visible in this tissue section. Thus, the tissue section represents the proximal and distal areas of the tissue. The lower panels at ×20 magnification (Fig. 5b) clearly show HSP70 induction in areas proximal to the microwave site due to direct microwave effect. Only low levels of HSP70 staining were detected in areas distal to the treatment site at the ends of the tissue (Fig. 5b). Examination of treated areas of the tissues revealed HSP70 expression in cells remaining in the treated area and in the area proximal to treatment (Fig. 5c). These data show that microwave treatment induces a localised heat shock response.

### Microwave treatment induces a cellular stress response

Cell stress leads to translational stress. HSP70 family members alter mRNA metabolism, including mRNA decay and translation.^36^ During stress, untranslated or translation-stalled cytoplasmic mRNAs are sequestered into stress granules.^37^ To investigate whether a stress response was induced by microwave energy, induction of G3BP a marker of cellular stress granule formation, was examined by immunofluorescence microscopy. In the absence of cellular stress, G3BP staining appears diffuse, however, under conditions of prolonged stress the staining becomes punctate. A mock-treated tissue was included in the analysis, as well as a positive control tissue that had been incubated at 40 °C in culture for 6 h to induce heat shock (Fig. 5d + ve control). The mock-treated tissue showed no G3BP staining (Fig. 5d Mock-treated) while G3BP was clearly induced due to heating in the positive control tissue (Fig. 5d + ve control). In microwave-treated tissues, distal regions showed low levels of G3BP staining (Fig. 5d “Distal”) but there was an increase in G3BP in the proximal and treated regions. Punctate staining consistent with cytoplasmic granules was visible in the cells at the edge of the proximal region (Fig. 5d, “Proximal”, white arrowheads) and in cells in the in the treatment site (Fig. 5d “Treated”, white arrowheads) indicating stress granule formation. Another stress granule protein, PABPC1 was examined in SiHa tissues treated with microwaves. G3BP and PABPC1 levels were both increased in the 16-h re-incubated tissues compared to 0 h post-treatment (Fig. 5e). There was clear cytoplasmic co-localisation of G3BP and PABPC1 at 0 h which was increased at 16 h post-microwave treatment (Fig. 5e). These data reveal that microwave treatment produces a robust heat shock response leading to translational stress. Since microwave treatment does not significantly inhibit HPV oncogene transcription at 16 h post treatment yet viral oncoprotein expression is reduced, our data suggest that the stress response due to microwave energy inhibits translation of HPV oncoproteins E6 and E7.

### Microwave treatment effects on in vitro-grown HPV16-infected precancer tissues

**Fig. 6:**
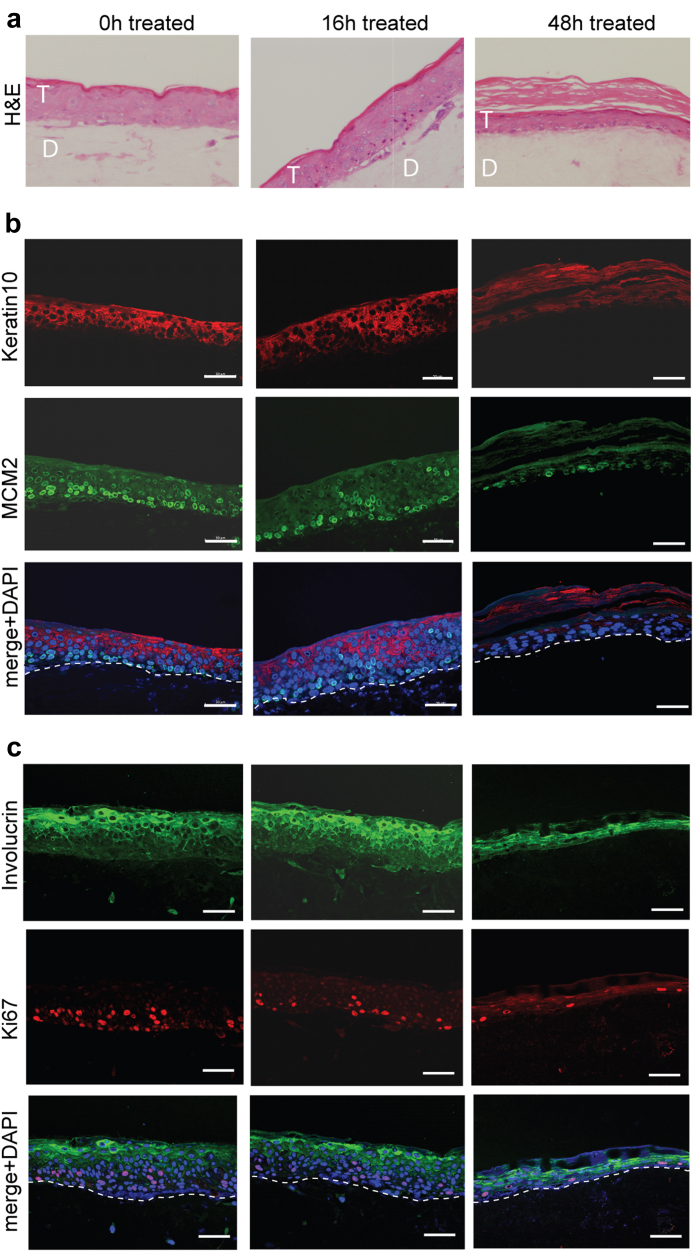
**Effect of microwave treatment on in vitro-grown 3D NIKS16 tissue**s. (a). H&E-stained sections of NIKS16 tissues microwave-treated and reincubated for 0, 16 and 48 h. T = SiHa tissue. D = collagen/fibroblast dermal equivalent. (b). Immunofluorescence staining of distal areas of microwave-treated NIKS16 tissues (microwave-treated and reincubated for 0, 16 and 48 h) with antibodies against epithelial differentiation marker keratin 10 (red staining) and cell proliferation marker MCM2 (Green staining). The bottom image in each column shows the merged images and nuclei stained with DAPI. (c). Immunofluorescence staining of distal areas of microwave-treated NIKS16 tissues (microwave-treated and reincubated for 0, 16 and 48 h) with antibodies against epithelial differentiation marker involucrin (green staining) and cell proliferation marker Ki67 (red staining). The bottom image in each column shows the merged images and nuclei stained with DAPI. White dotted lines show the basal layer of the tissues and junction with the dermal equivalent. Scale bars = 50 μM. Data shown are representative images from three separate experiments.

Microwave therapy may prove most useful to treat HPV-positive precancers to inhibit cancer progression. Therefore, next we tested the effect of microwave energy on 3D cultures of NIKS16 cells, a model of HPV16-positive anogenital intraepithelial neoplasia.^23^ All the images in Fig. 6 are of distal areas of microwave-treated tissues but the proximal and treated areas of replicate tissues showed similar cell proliferation and differentiation changes. Microwave treatment resulted in marked structural changes to the tissue with increased keratinisation at 48 h post-treatment (Fig. 6a). A reduction in expression of MCM2 and Ki67 over 48 h indicated decreased cell proliferation (Fig. 6b and c). This was accompanied by an increase in expression of differentiation markers keratin 10 and involucrin (Fig. 6b and c). These data reveal a rebalancing from proliferation to differentiation due to microwave treatment. A similar effect was seen in HPV18-positive 3D tissue cultures (Supplementary Figure S2). Expression levels of E6 and E7 oncoproteins are too low to detect efficiently in NIKS16 cells by antibodies. Therefore, we were unable to measure loss of viral oncoprotein expression in these tissues. However, the increased differentiation noted in microwave-treated tissues suggests an abrogation of E6 and E7 expression since these proteins inhibit differentiation in infected tissues.^29^^,^^30^

**Fig. 7:**
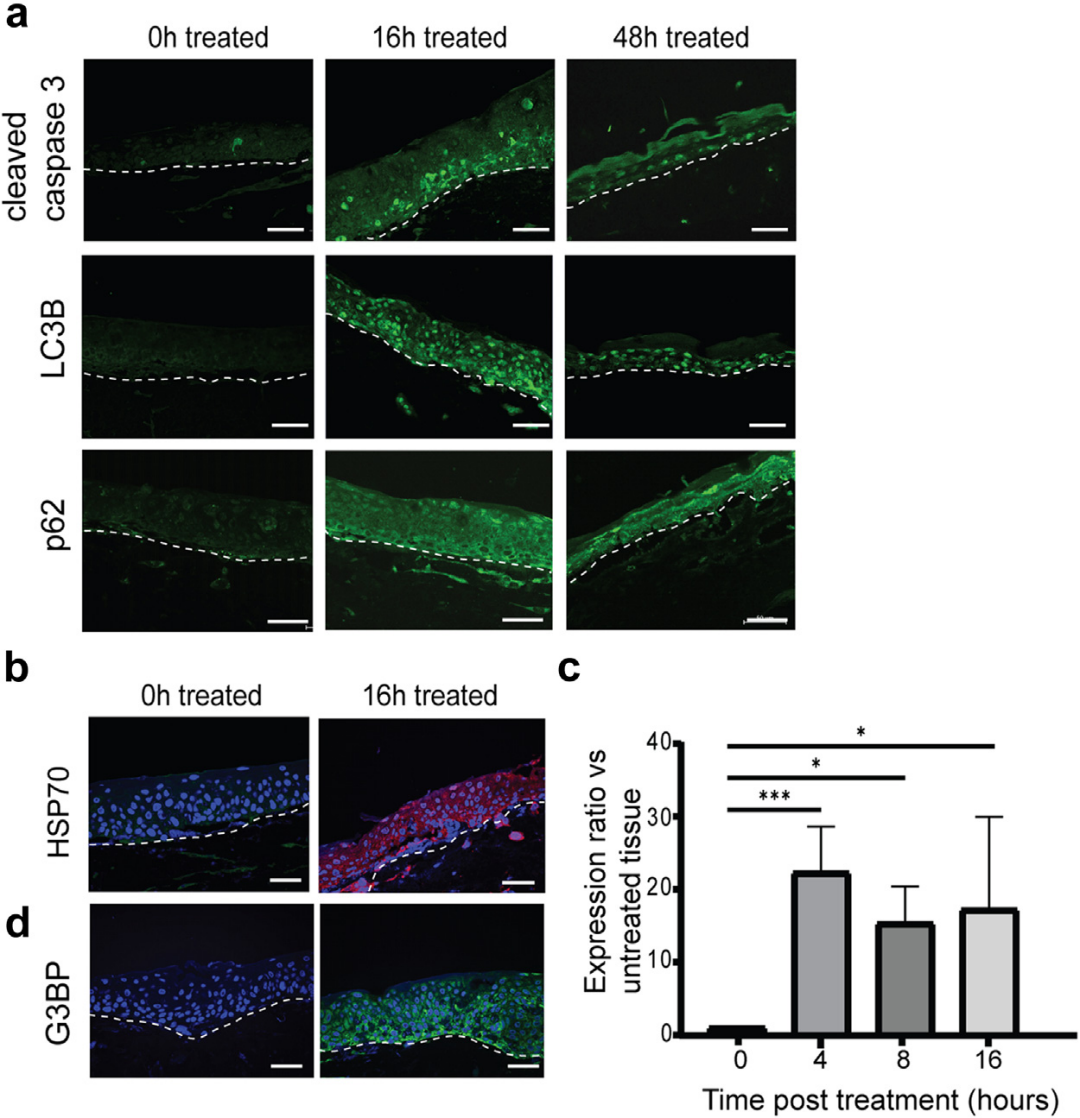
**Microwave treatment results in apoptosis and autophagy and induction of a heat shock response.** (a). Immunofluorescence staining (green staining) of microwave-treated NIKS16 tissues (microwave-treated and reincubated for 0, 16 and 48 h) with antibodies against cleaved caspase 3, LC3B and p62. (b). Immunofluorescence staining of microwave-treated NIKS16 tissues (microwave-treated and reincubated for 0 and 16 h) with an antibody against HSP70 (red staining). (c). Increase in HSP70 mRNA expression in NIKS16 3D tissues over time following microwave treatment and reincubation. ∗∗∗p < 0.001. ∗p < 0.05. p-values were determined using student's t-tests. (d). Immunofluorescence staining of microwave-treated NIKS16 tissues (microwave-treated and reincubated for 0 and 16 h) with an antibody against and G3BP (green staining). Nuclei are counterstained with DAPI. White dotted lines show the basal layer of the tissues and junction with the dermal equivalent. Scale bars = 50 μM. Data shown are representative images from three separate experiments.

Like SiHa tissues, microwave treatment of NIKS16 tissues induced sustained expression of cleaved caspase 3, and the autophagy markers LC3B and p62 (Fig. 7a). Finally, we confirmed that microwave treatment induced HSP70 (Fig. 7b). Induction of HSP70 expression was rapid, within the first 4 h of treatment, and was sustained for 16 h post-treatment (Fig. 7c). This resulted in translation stress in the NIKS16 tissues because levels of the stress granule marker G3BP were increased in the cytoplasm of treated cells (Fig. 7d). These data confirm the cellular response to microwave treatment in a different type of HPV-infected tissue.

## Discussion

The microwave treatment protocol that we developed resulted in precise tissue disruption at the target site. The dimensions of the area of treatment in SiHa cells agreed well with those measured *in vivo* in the previous plantar wart study and there was no significant spreading into the dermal equivalent layer as expected due to the limited depth of penetration of microwave energy.^18^ Cellular apoptosis was detected in areas proximal to the treatment site. Induction of apoptosis in the tissues is in agreement with another study where microwave treatment was shown to induce caspase-dependent apoptosis in a leukaemic cell line.^38^ Apoptosis was sustained over at least 72 h and was also observed at sites distal to the treatment site, but at a lower level than that observed at proximal sites. Autophagy markers LC3B and p62 were upregulated upon heat shock in both SiHa (data not shown) and NIKS16 tissues. Following re-incubation at 37 °C after microwave treatment, cells in the area outside of the treatment site in SiHa tissues continued to grow over a 6-day period but there was evidence of reduced proliferation as shown by reduced expression of Ki67 compared to mock-treated tissues. Importantly, no regrowth in the treated area was observed. Taken together, the data suggest that microwave energy could be used to precisely treat anogenital lesions, kill HPV-infected tumour cells and inhibit regrowth from the surrounding area.

The heat shock response is a normal response of cells to ensure survival under conditions of environmental stress. As expected, microwave treatment induced a localised heat shock response due to hyperthermia.^34^ Of all the heat shock chaperone proteins, HSP70 is the most strongly induced by cell stress and it acts to modulate the structure of misfolded proteins that accumulate as a direct result of cell stress.^35^ In microwave-treated tissues, HSP70 was specifically and rapidly induced in and around the treatment site. This emphasises the locus-restricted nature of microwave-induced hyperthermia. The heat shock response also induces formation of stress granules which contain mRNA/protein assemblies from stalled translation initiation events.^37^ Following adverse cellular events, stress granules can be remodelled to allow re-initiation of protein translation or to allow autophagy.^36^ Analysis of G3BP and PABPC1, revealed stress granule formation particularly in cells of the treated and proximal tissue areas following microwave treatment. Therefore, we suggest that microwave hyperthermia leads to heat shock and translational stress followed by cell death at the treatment site, but the cell stress response is muted at sites distal to the treated area. While we found that E6E7 bicistronic mRNA expression was unaltered upon microwave treatment, except at later time points, there was a statistically significant decrease in E6 and E7 protein expression. This result implies that heat regulation of E6 and E7 expression occurs at a post-transcriptional level, possibly at the level of translational arrest and sequestration of the E6/E7 mRNAs into stress granules.^37^

In HPV-positive cancer cells such as SiHa cells, E6 forms a complex with p53 and E6AP leading to p53 proteasomal degradation.^25^^,^^33^ Cellular heat shock increases expression of p53 and leads to its phosphorylation and subsequent tetramer formation in the nucleus. This results in stabilisation of p53^39^, ^40^, ^41^, ^42^ which can then activate transcription of HSP70, leading to the heat shock response and apoptosis.^43^ Our data suggest a heat-shock-induced reduction in E6 protein levels, which should add to the increased p53 levels due to hyperthermia. Taken together, these dual control mechanisms explain the rapid and significant p53 upregulation detected upon microwave treatment at the treatment site, and in areas proximal to the treatment site. A similar result in conventional cell culture of SiHa cells was reported previously where hyperthermia caused loss of E6, preventing p53 degradation. In that study, de novo synthesis of p53 was also detected and this led to the normal induction of apoptosis in HPV-positive cancer cells.^44^ A therapy which induces p53 and apoptosis of cervical cancer cells shows promise for anticancer therapy.

As well as influencing the E6/p53 axis, microwave treatment resulted in upregulated levels of the E7 target protein Rb. Normally Rb controls the transition from G1 to S phase of the cell cycle. Phosphorylation of Rb by cyclin dependent kinases allows release of E2F transcription factor to promote transcription of cell cycle-related genes. HPV E7 binds Rb and releases E2F to activate expression of cell cycle-related genes.^26^ Thus, loss of E7 due to hyperthermia should repress cell proliferation and inhibit G1 to S-phase cell cycle progression. Our observation of decreased levels of S-phase-specific proteins MCM2 and Ki67 upon microwave treatment supports this conclusion. However, under conditions of cell stress, p38 stress-activated protein kinase represses E2F through selective phosphorylation of Rb and disallowing its cyclin-dependent kinase phosphorylation. p38-phosphorylated Rb now has increased affinity for E2F. This results in downregulation of transcription of E2F-regulated cell cycle-related genes and inhibition of cell-cycle progression.^45^ Therefore, although E7 levels may be repressed in microwave-treated cells, Rb can no longer activates cell proliferation due to the cell stress response.

In summary, the effects of E6 and E7 reduction in microwave-treated cells may be enhanced by the manner in which p53 and Rb respond to cell stress. Since HPV-associated cancer progression is initiated and sustained by increased expression of the viral oncoproteins E6 and E7, our data suggest that microwave treatment could reverse the tumour phenotype of cervical cancer cells.

Hyperthermia can cause HSP70 to be released into the environment.^46^ HSP70 is a DAMP which can activate antiviral and antitumoural pathways.^10^ Cell-released HSP70 can bind antigen presenting cells,^47^^,^^48^ but as a chaperone, HSP70 can be released bound to tumour cell antigens. This results in uptake and presentation of the antigens by antigen presentation cells leading to induction of anti-tumour CD8^+^ T-cell responses.^49^ Therefore, microwave treatment has potential to stimulate cell-mediated immunity. This is particularly relevant in the case of treatment of HPV-associated lesions since stimulation of the immune response could clear the virus and prevent reinfections. In the plantar wart study, microwaves potentiated cutaneous immunity to HPV^18^ while in studies of genital warts, hyperthermia upregulated APOBEC antiviral activity^50^ and induced a range of proteins involved in antiviral responses.^51^ Immune activation could result in clearance of lesions not only at the treatment site but at other, untreated sites as suggested in a study of hyperthermia to treat genital warts.^50^ This possibility requires further investigation of microwave therapy against HPV-associated cancers and precancers *in vivo*.

Compared to ablative procedures such as cryo- or laser therapy, microwave therapy can have a greater depth of penetration. Moreover, it produces no vapour or smoke^52^ and so is safe for resolution of virus-positive lesions. The small size of the treated area is also advantageous and different device probes can be manufactured to fit particular clinical uses. In the *in vivo* verruca and actinic keratoses studies, participants experienced pain from microwave treatment but it was bearable and transient.^18^^,^^19^ These studies support favourable therapeutic effects *in vivo*, particularly for the localised nature of the treatment. Although treatment of mucosal tissue *in vivo*, as opposed to cutaneous sites could result in a higher level of pain, this issue could be solved using local anaesthesia. If microwave treatment induced antiviral or antitumoural immunity, local recurrence could be alleviated while lesions at untreated sites might regress.

The most likely clinical use for a microwave device is in treatment of genital warts and anal and vulvar precancers and cancers, especially where multifocal disease occurs. The microwave device is currently CE marked for dermatology use and so cannot currently be used to examine efficacy in the clinic in treating these diseases. Although it has allowed us to understand the molecular basis of microwave-induced tissue apoptosis, the *in vitro* nature of our study is a major limitation. The nature of microwave heating in various tissues is known to be different such as liver, warts or actinic keratosis^15^^,^^18^^,^^19^ where the power and time are adjusted to suit the desired therapeutic envelope. These adjustments can be model led in computer simulation prior to conducting treatment of any new site. However, any *in vivo* study on anogenital lesions would require careful assessment of treatment regimes, treatment tolerance and efficacy for each type of lesion. It will be very important to monitor any disease recurrence over time, a factor that has not yet been analysed in the published *in vivo* studies.

In conclusion, our data show that inhibition of cell proliferation and induction of apoptosis in HPV-positive cervical tumour tissues can be induced by hyperthermia delivered in a precise, highly localised manner by microwaves. The mechanism of cell death is through activation of the cellular stress response and repression of HPV oncoprotein expression. Precision microwave delivery may present a potential new treatment for HPV-positive anogenital precancerous lesions and cancers.

## Contributors

All authors have read and approved the final version of the manuscript.

MC: investigation, data interpretation, drafting the manuscript. Verified the underlying data.

IE: investigation, data analysis. Verified the underlying data.

AK: investigation, data analysis. Verified the underlying data.

AS: investigation, data analysis, project administration.

SVG: conceptualisation, funding acquisition, supervision, visualisation, writing the manuscript. Verified the underlying data.

## Data sharing statement

All data generated or analysed during this study are included in this report, and are available upon request from the corresponding author. All materials are commercially available.

## Declaration of interests

Emblation Ltd (Alloa, Scotland) provided the microwave device. Emblation Ltd had no input into experimental design, data collection or analysis. We declare no conflict of interest.
