# Supplementary material for: Microwave hyperthermia represses human papillomavirus oncoprotein activity and induces cell death due to cell stress in 3D tissue models of anogenital precancers and cancers
Source: eBioMedicine. 2023 Apr 15;91:104577. doi: 10.1016/j.ebiom.2023.104577 (PMC10130467; doi:10.1016/j.ebiom.2023.104577)
Supplement: Supplementary Figures S1–S2 and files [file mmc1.pdf]

SUPPLEMENTARY MATERIAL

Conley et al. Microwave hyperthermia represses human papillomavirus oncoprotein activity and induces cell death due to cell stress in 3D tissue models of anogenital precancers and cancers.

Table of Contents

Page Number	Content
Page 2.	Supplementary Figure 1
Page 3.	Supplementary Figure 2
Page 4.	Captions for supplementary figures
Page 5.	Antibody validation file
Page 6-8.	Cell line check list file

Supplementary Figure 1: Experimental set-up for microwave treatment of 3D tissue cultures.

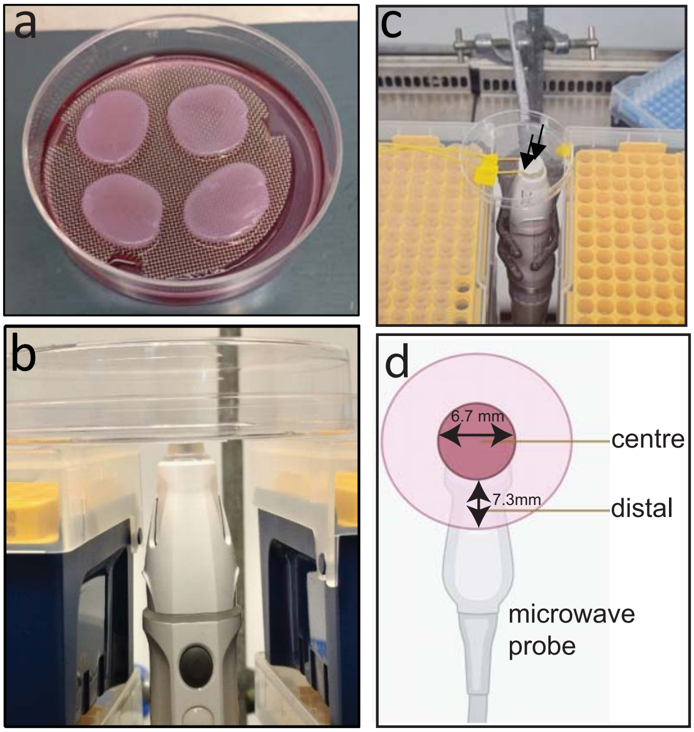

Supplementary Figure 2: Effect of microwave treatment on HPV18-positive 3D tissue cultures

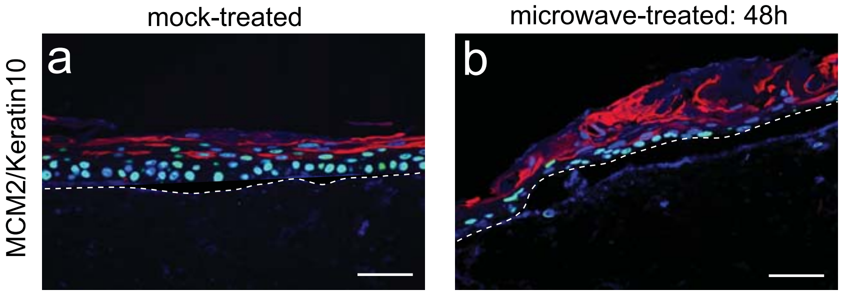

Captions for supplementary material

Supplementary Figure 1: Experimental set-up for microwave treatment of 3D tissue cultures. (a). Four 3D SiHa tissues growing on a metal grid at the air-liquid interface representing two control and two test tissues. Replicate experimental data were collected from separate experiments. (b). Tissues were transferred onto the lid of a 6 cm^2 dish with the outer epidermal surface in contact with the plastic and placed above the microwave device. A side of view of the experimental setup is shown. (c). A top view of the experimental set up showing the placement of the temperature sensor (yellow horizontal probe and arrowed).

Supplementary Figure 2: Effect of microwave treatment on HPV18-positive 3D tissue cultures. 3D tissues grown from normal human immortalised keratinocytes stably transfected with HPV18 genomes. (a). mock-treated tissues. (b) tissues treated with microwaves followed by incubation for 16 hours. White dotted lines show the basal layer of the tissues and junction with the dermal equivalent. Scale bars = 50 μM

Antibody validation

Antibody	RRID	Supplier	Catalogue#	Dilution (technique)
Cleaved caspase 3	RRID:AB_2243952	R&D Systems	AF835	1:1000 (IHC)
				0.3 µg/ml (IF)
MCM2	RRID:AB_881276	Abcam	ab31159	1:100 (IHC, IF)
Ki67	RRID:AB_2142367	Agilent	M7240	1:200 (IHC)
Ki67	RRID:AB_2861195	Abcam	ab206633	1:1000 (IF)
p53	RRID:AB_297667	Abcam	ab1101	1:3000 (IHC)
Rb	RRID:AB_823629	Cell signaling Technologies	9309	1:1600 (IHC)
HSP70	RRID:AB_477057	Sigma-Aldrich	H5147	1:3200 (IHC)
				1:200 (IF)
HPV E6	RRID:AB_2933993	Euromedex	6F4	1:200 (IF)
HPV E7	RRID:AB_2533057	Thermo Fisher	28-0006	1:50 (IF)
G3BP	RRID:AB_941699	Abcam	ab56574	1:250 (IF)
PABPC1	RRID:AB_777008	Abcam	ab21060	1:1000 (IF)
Keratin 10	RRID:AB_306950	Abcam	ab9026	1:200 (IF)
Involucrin	RRID:AB_477129	Sigma-Aldrich	I9018	1:200 (IF)
LC3B	RRID:AB_10003146	Novus	NB100-2220	1:200 (IF)
p62	RRID:AB_1953130	MBL International	PM045B	1:500

Table 1. details of antibodies used in the study. IHC, immunohistochemistry. IF, immunofluorescence.

Antibody validation was carried out by

(a) examining protein detection at a range of antibody concentrations.
(b) quantifying reproducibility of protein detection between experiments using western blotting.
(c) specificity was examined by carrying out western blotting to ensure that a single band of the correct molecular mass was obtained with each antibody.
(d) Secondary antibody only controls were included in every experiment.
(e) E6 and E7 antibodies were validated by comparing reactivity in immunofluorescence in HPV-negative C33a cells and C33a cells stably expressing E6 or E7.
(f) Involucrin and keratin 10 antibodies were validated by comparing reactivity in undifferentiated NIKS cells (which do not express these proteins) with the same cells induced to differentiated (when these proteins are expressed).
(g) Other antibodies were verified by comparing protein depletion following siRNA knock down by western blotting.
(h) The same batch of each antibody was used throughout.

Cell Line Checklist for Manuscripts and Grant Applications

This checklist is a resource for scientists who write or review manuscripts and/or for grant applications that use cell lines. Cross-contaminated cell lines could give unreliable results if used for research because they no longer correspond to the reported donor tissue and so may not represent the correct species, tissue type or disease state. Such misidentified or false cell lines produce unreliable research data and we urge reviewers to highlight their use wherever possible.

This checklist will help the author or reviewer to look for obvious cell line quality concerns. The checklist may also be used to communicate any quality concerns to be addressed prior to publication or funding.

Manuscript or Grant Information

Title or Manuscript/Grant ID:	Microwave hyperthermia represses human papillomavirus oncoprotein activity and induces cell death due to cell stress in 3D tissue models of anogenital precancers and cancers.
Cell Lines used:	SiHa<br>NIKS16
Cell Lines used with Quality Concerns:	NONE

Cell Line Information

**Reporting Requirement**	**Indicate "Yes" or "No" (No includes Not Known)**<br>**Add further comment if required**
Cell line is known to be cross-contaminated or otherwise misidentified:<br>See the ICLAC website for a register of known misidentified cell lines and Recommendation 1) below.	No
Authentication testing has been performed:<br>The method and results should be listed.<br>See Recommendation 2) below.	SiHa: HPV genome quantification and HPV E6E7 mRNA expression quantification checked as unchanged by qRT-PCR. Morphology in 2D and 3D culture verified as unchanged in every experiment. SiHa cells are non-invasive and invasion was not observed in any 3D tissue culture experiment.<br><br>NIKS16/NIKS18: HPV genome quantification and HPV E6E7 and E4 mRNA expression quantification was checked by qRT-PCR for any changes from batch-to-batch cell growth. Tissue distribution of cellular proliferation and differentiation markers was validated in every experiment by examining in-tissue expression of MCM2, Ki67, Involucrin and Keratin 10, and viral E4 and L1 protein expression (validating active HPV replication) in untreated tissues and in 2D culture. No changes in HPV genome numbers, mRNA expression or protein expression were detected.

Human cell lines: STR profile is available with the manuscript/grant application:<br>See Recommendation 2) below.	No, the validation above is considered sufficient.
Mycoplasma testing has been performed:<br>The method and results should be listed.	Yes, using MycoAlert®Mycoplasma Detection Kit (Lonza cat No. LT07-318) according to the manufacturer's instructions.
Source for cell line is listed:<br>The catalogue number should be included if obtained from a cell line repository.<br>See Recommendation 3) below.	SiHa: ATCC #HTB-35<br>NIKS16/NIKS18<br>Flores, E. R. et al. Establishment of the human papillomavirus type 16 (HPV-16) life cycle in an immortalized human foreskin keratinocyte cell line. *Virology* **262:**344–54 (1999). doi: 10.1006/viro.1999.9868.<br>Isaacson Weschler E, et al. Reconstruction of human papillomavirus type 16-mediated early-stage neoplasia implicated E6/E7deregualtion and the loss of contact inhibition in neoplastic progression. *J Virol* **86**:6358–6364 (2012). doi.org/10.1128/JVI.07069-1
RRID Number for cell line is listed:<br>The Resource Identification Initiative (RRID) is meant to help researchers cite the important resources used in scientific papers.<br>See Recommendation 4) below.	SiHa: IZSLER Cat# BS TCL 112, RRID:CVCL_0032<br>NIKS16: RRID:CVCL_B0UM<br>NIKS18: RRID:CVCL_B0UP
Sufficient information is given to replicate experiments using the cell line:<br>See Recommendation 5) below.	SiHa cells are grown under standard conditions for cervical cancer cells lines.<br>NIKS16/18 are grown as primary keratinocytes on J2 3T3 fibroblast feed layers in E-medium as previously described: Jeon S, Allen-Hoffman BL, Lambert PF. Integration of human papillomavirus type 16 into the human genome correlates with a selective growth advantage of cells. *J Virol* **69**: 2989-97 (1995).<br>Passage numbers are kept to <15 passages to limit tumour progression in vitro. Cell stocks are frozen after every passage.

Recommendations

1) ICLAC recommends that false cell lines (misidentified cell lines with no known authentic stock) should not be used. ICLAC's register of misidentified cell lines can be found at http://iclac.org/databases/cross-contaminations.

2) ICLAC recommends that authentication testing should always be performed on established cell lines regardless of the application; the test method and results should be included in the Materials and Methods section. Testing should be done, at minimum, at the beginning and end of experimental work.

   For human cell lines, short tandem repeat (STR) profiling should be performed and compared to results from donor tissue, or to online databases of human cell line STR reference profiles.

   More information can be found in the published Standard: ANSI/ATCC ASN-0002-2011 Authentication of Human Cell Lines: Standardization of STR Profiling. ANSI eStandard Store.

   For non-human cell lines, best practice will vary with the species being tested. At minimum, species should be confirmed using an appropriate method such as karyotyping, isoenzyme analysis, or mitochondrial DNA typing (DNA barcoding).

More information on authentication testing can be found at http://iclac.org/references/.

3) It will be helpful for the reader if authors can include a reference, to provide more information on the cell line's establishment and characterization. However, not all cell lines have this information available in the public domain.

4) Cell line RRIDs are assigned through a collaboration between Cellosaurus and the Resource Identification Initiative. RRIDs can be found by searching for cell lines at https://web.expasy.org/cellosaurus/

5) This information may include the growth medium used, including additives; any additional growth requirements, including special substrates and gas mixtures; and the passage number or population doubling level (PDL) used for experimental work.

   Passage number is important when working with early passage or finite cultures, or cell lines where changes in phenotype have been documented with increasing passage. ICLAC recommends that laboratories freeze down stocks when they first receive a cell line and set a limit (e.g. 20 passages) to avoid overpassaging. More information can be found at http://iclac.org/resources/advice-scientists/

Notes or Further Comments

Please see the following publications for additional validation of NIKS16 cells.

Human Papillomavirus E2 Regulates SRSF3 (SRp20) To Promote Capsid Protein Expression in Infected Differentiated Keratinocytes. Klymenko T, Hernandez-Lopez H, MacDonald AI, Bodily JM, Graham SV. J Virol. 2016 Apr 29;90(10):5047-58. doi: 10.1128/JVI.03073-15

RNA-Seq Analysis of Differentiated Keratinocytes Reveals a Massive Response to Late Events during Human Papillomavirus 16 Infection, Including Loss of Epithelial Barrier Function. Klymenko T, Gu Q, Herbert I, Stevenson A, Iliev V, Watkins G, Pollock C, Bhatia R, Cuschieri K, Herzyk P, Gatherer D, Graham SV. J Virol. 2017 Nov 30;91(24):e01001-17. doi: 10.1128/JVI.01001-17

Human papillomavirus type 16 infection activates the host serine arginine protein kinase 1 (SRPK1) - splicing factor axis. Mole S, Faizo AAA, Hernandez-Lopez H, Griffiths M, Stevenson A, Roberts S, Graham SV. J Gen Virol. 2020 May;101(5):523-532. doi: 10.1099/jgv.0.001402
